# Blended (online and in‐person) Women’s Health Interprofessional Learning by Simulation (WHIPLS) for medical and midwifery students

**DOI:** 10.1111/ajo.13531

**Published:** 2022-04-18

**Authors:** Timothy Lee, Si Woo Yoon, Shavi Fernando, Suzanne Willey, Arunaz Kumar

**Affiliations:** ^1^ Department of Obstetrics and Gynaecology Monash University Clayton Victoria Australia; ^2^ Faculty of Medicine, Nursing and Health Sciences Monash University Clayton Victoria Australia; ^3^ Monash Nursing and Midwifery Monash University Frankston Victoria Australia

**Keywords:** pedagogy, simulation training, obstetrics, gynaecology, medical students

## Abstract

**Background:**

Blended teaching combines traditional in‐person components (simulation‐based training and clinical‐based placement) with online resources. Due to the COVID‐19 pandemic, we modified our Women's Health Interprofessional Learning through Simulation (WHIPLS) program – to develop core obstetric and gynaecological skills – into a blended teaching program. There is limited literature reporting the observations of blended teaching on learning.

**Aims:**

To qualitatively evaluate the blended teaching program and explore how it contributes to learning.

**Materials and Methods:**

This study was performed at Monash University in Melbourne, Australia. A total of 98 medical students and 39 midwifery students participated. Data were collected by written survey and analysed by authors using a thematic analysis framework.

**Results:**

Students reported that in‐person teaching remains a vital aspect of their curriculum, contributing an averaged 63.2% toward an individual's learning, compared with online. Five substantial themes demonstrate how students learnt and maximised education opportunities using a blended teaching program: ‘low‐pressure simulation environments’, ‘peer‐assisted learning’, ‘haptic learning’, ‘scaffolded learning’ and ‘the impact of online discourse’.

**Discussion:**

In‐person teaching remains a cornerstone of obstetric and gynaecological clinical skills education, of which interprofessional simulation and clinical‐based placement are key components. Teaching via online discourse alone, is not sufficient to completely replace and provide comparable learning outcomes, but certainly plays an important role to prime students' learning and to maximise in‐person opportunities and resources. Our study reveals key pedagogies of a blended (online and in‐person) learning program, providing further evidence to support its ongoing utility as a feasible and warranted approach to learning.

## INTRODUCTION

Historically, medical student education in obstetrics and gynaecology has relied on bedside teaching, where real‐time, real‐life experiences might intimidate both the learner and the patient. Moreover, the intimate nature of obstetric or gynaecological examinations often requires the clinician to be proficient and able to build rapport with the patient, both of which are skills that students may not yet have attained. This has led to increased use of simulation‐based training that allows students to practice in a controlled environment under the supervision of tutors.^1^, ^2^ Well‐designed simulation enhances learning outcomes in medicine, without simultaneously compromising the safety of patients.^3^

Such simulation‐based training can be performed within interprofessional settings to foster teamwork, better recognise the complimentary nature of roles, and achieve common learning objectives.^4^ In clinical settings, clarifying expectations between midwifery preceptor and medical student impacts on learning experiences.^5^ Previously, there has been introduction of the Women's Health Interprofessional Learning through Simulation (WHIPLS) program for medical and midwifery students to develop core obstetrics and gynaecology clinical skills.^6^ This involves pre‐reading materials and lecture‐based orientation, prior to attending an in‐person clinical skills workshop to learn how to perform speculum and vaginal examinations, and manage the second and third stage of labour. Reported outcomes from this program included an increase in positive attitudes toward interprofessional simulation‐based education, a better understanding of the role simulation has in learning, reflection on professional identity, and development of respect for each profession as equals.^7^

The COVID‐19 era has affected various aspects of the healthcare system including medical student education.^8^ Throughout 2020/21, clinical placements were cancelled or postponed due to hospital exposure risk, and there continues to be a significant shift toward tutorials and lectures being delivered online. Consequently, a modified WHIPLS program was created by transitioning all lectures to be pre‐recorded and made available online, and by incorporating an online demonstration and live Q&A session prior to attending the workshops. With the experience gained, we envisage that blended teaching (a combination of online and in‐person) will remain a key component of healthcare education – whereby educators provide as much as can be achieved online, while maintaining important aspects of in‐person, hands‐on, clinical learning.

There is limited literature currently reporting the observations and effects that blended teaching has on learning. This study qualitatively evaluates a blended program for teaching core obstetrics and gynaecology clinical skills, and asks the question, ‘how does a combination of online (pre‐reading and resources, live demonstration and Q&A) and in‐person teaching (clinical skills workshop, clinical‐based placement) contribute to learning?’ – with the aim to provide insight into how students learn and maximise educational opportunities using these approaches.

## MATERIALS AND METHODS

### Design, setting and participants

Using a constructivist approach, we sought to explore the experiences and perceptions of our study participants, medical and midwifery students, undertaking the modified WHIPLS program. Constructivism, an educational theory, is the notion that further knowledge is built by integrating one's personal learning experiences with foundational knowledge they already have.^9^, ^10^ Furthermore, it proposes that learning is an active process, a social activity and occurs in a contextual manner. As reflected by the WHIPLS program, teachers act as facilitators and encourage shared authority and knowledge.

**Figure 1:**
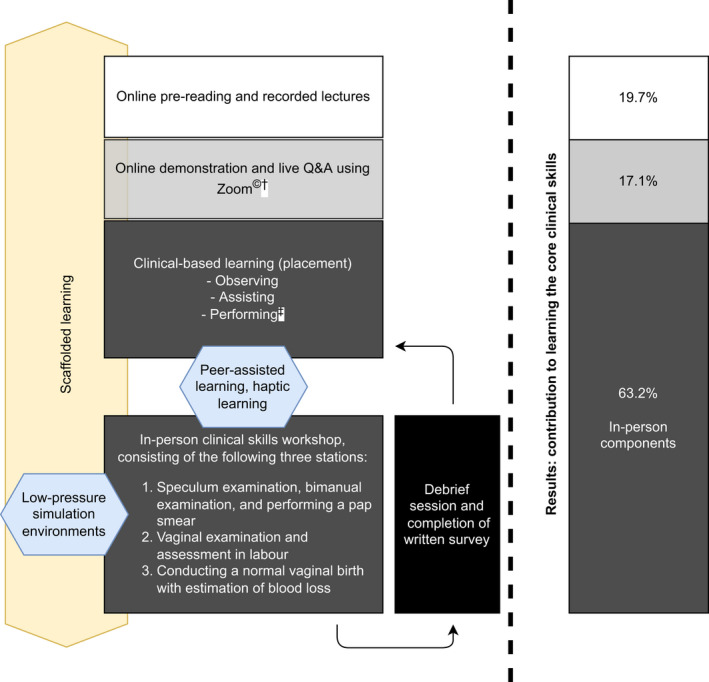
(Left) Modified Women's Health Interprofessional Learning through Simulation (WHIPLS) program; (right) results of contribution to learning core clinical skills. ^†^Zoom Video Communications, Inc., San Jose, California. ^‡^Students were only credentialed to perform procedures after completion of the workshop.

The study was performed at a tertiary hospital‐based clinical school in Melbourne, Australia. Medical students were in their penultimate year of medical school, and the obstetrics and gynaecology term ran over 9 weeks. The program is detailed in Figure 1. Medical students were provided access to the online resources at commencement of their term, with the online simulated demonstration and live Q&A taking place at the end of their first week of term. This was followed by clinical‐based placement, with the in‐person clinical skills workshop held midway through their term. Third‐year midwifery students were allocated to the program evenly over the duration of their clinical year. Similarly, they received access to online resources a week prior to the online demonstration and in‐person workshop. Administrative staff filmed the online demonstration and Q&A on a smartphone camera via Zoom^©^. Common learning outcomes were collaboratively set by medical and midwifery curriculum leads.

### Ethics

The study was approved by the Monash University Human Research Ethics Committee (Project/Reference Number 24832). Participants provided written consent releasing their deidentified responses for research purposes. All participants who attended the WHIPLS program were invited to participate in the study which required them to respond to a survey.

### Data collection

Across six independent sessions, a total of 98 medical and 39 midwifery students participated in the study. Data were collected by paper survey (see Appendix 1), performed immediately after the in‐person clinical skills workshop (*n* = 137). Students were reassured their responses would not affect their university outcomes. Reponses were collected and tabled into an Excel spreadsheet verbatim. The average length of response to each question was several sentences.

### Team reflexivity

Diversity among the authors provided a rigorous and triangulated interpretation of the data, contributing differing perspectives and insights given their diverse hierarchical status, experience, knowledge, and clinical background. TL is a postgraduate third‐year junior doctor working in obstetrics and gynaecology and is a tutor for the WHIPLS program. SY is a fourth‐year undergraduate medical student who undertook the modified WHIPLS program as part of their university curriculum but was not enrolled as a study participant. SF is a consultant obstetrician and gynaecologist and coordinator of the medical curriculum. SW is a senior midwifery lecturer in nursing and midwifery education and research. AK is a senior obstetrician and gynaecologist, and an academic health education researcher with experience in qualitative research.

### Analysis

Based on Braun and Clarke,^11^ a thematic analysis framework was established by all authors prior to addressing the data. TL and SY independently performed initial coding. A reflexive inductive approach was used to allow for greater realisation and conceptualisation of the data. The authors then identified meaning‐based patterns to construct candidate themes, or in instances, promoted substantial codes to themes themselves. In consultation with AK, the authors then compared, contrasted, and negotiated their candidate themes. After several rounds of analysis, a consensus on the results was reached.

## RESULTS

### Contribution to learning the core clinical skills

Students reported that in‐person teaching remains a vital aspect of their curriculum, with a reported averaged contribution of 63.2% of an individual student's learning coming from in‐person rather than online teaching (see Fig. 1).

**Figure 2:**
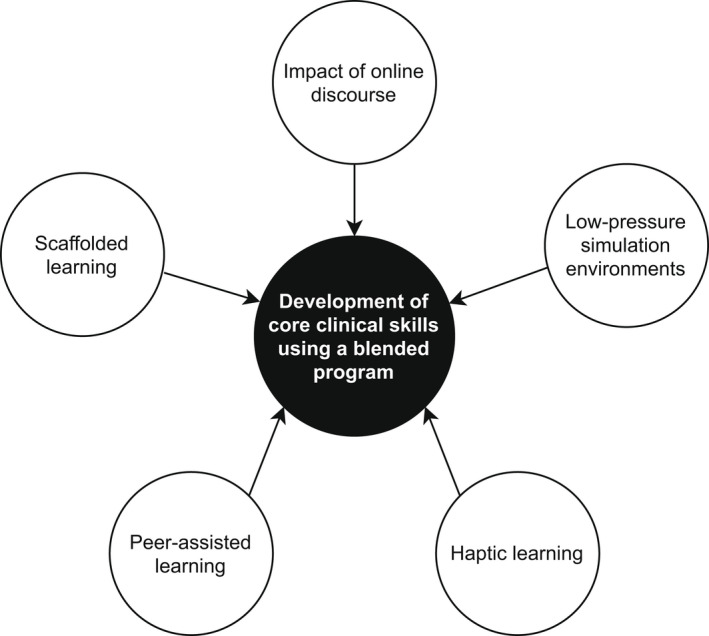
Five themes of pedagogy leading to development of core clinical skills using a blended program.

Five substantial themes demonstrate how students learnt and maximised educational opportunities within the modified WHIPLS blended teaching program (see Fig. 2). Dominant themes of ‘low‐pressure simulation environments’, ‘peer‐assisted learning’ and ‘haptic learning’ were identified when discussing the in‐person components, compared to themes of ‘scaffolded learning’ and ‘the impact of online discourse’ when discussing the online components of the program. Each of the five themes is presented below. Participants’ quotes endorsing each theme are presented in Table 1, with bracketed numbers given in the text to reference subthemes and relevant quotes.

**Table 1:** Themes and subthemes from the survey

1. Low‐pressure simulation environments	1.1 Removal of time pressures	‘… allows us to actually take our time practising.’ [Medical student, ME 47]
		‘Really great opportunity to practise skills slowly.’ [Midwifery student, MI 9]
	1.2 Removal of patient considerations	‘It was fantastic to practise in a stress‐free environment where I wasn't concerned about the awkwardness and stress of practising on a real‐life woman. It was non‐judgemental, I could take my time, and there wasn't the possibility of hurting anyone. It made me more confident to actually try things properly.’ [ME 77]
		‘The advantage of the model is that you could be comparatively ‘rough’ compared to a real patient. This was especially helpful for the intrapartum VE where it was difficult to feel the ‘V’ of the [lambdoid sutures].’ [ME 31] ‘… we can make our mistakes on models before attempting the exams on patients.’ [ME 42]
		‘… allowed me to practise without being worried about hurting the patients. I could also have a better feel and develop some muscle memory, giving me some confidence to try on real patients in the future.’ [ME 48]
		‘[There was] less concern with being wrong… since there are no complications for the woman.’ [ME 99]
	1.3 Access to focused, non‐judgemental expert feedback	‘… got a chance to ask lots of clarifying questions on my previous learning.’ [ME 71]
		‘… being able to troubleshoot before being in a clinical situation.’ [ME 72]
	1.4 Repeat attempts	‘… able to do things more than once. Easier to practise techniques and get immediate feedback.’ [ME 29]
		‘… given an opportunity to practise multiple times with feedback whilst having my questions answered.’ [ME 45]
2. Peer‐assisted learning	2.1 Interprofessional model	‘It was really useful to practise in groups of three… also helpful to have nursing students around to provide their own knowledge as well.’ [ME 97]
		‘Chatting to medical students. Learning different perspectives.’ [MI 39]
		‘Interaction with midwifery students was helpful. Having doctors to bridge the gap between sim and real patients was super helpful.’ [ME 93]
		‘Great way to ask questions in a large group.’ [ME 3]
		‘Incredibly helpful to speak [to] and observe the midwives.’ [ME 21]
	2.2 Group work	‘… [it was an] opportunity to practise skills, make mistakes, see what other people are doing and learn visually.’ [MI 3]
		‘Discussing with other students was more convenient.’ [ME 41]
		‘I was able to see many of them and was able to observe different people perform the same exam. This allowed me to explore and learn different techniques and taught me the most appropriate way I could perform the exam.’ [ME 37]
3. Haptic learning	3.1 Haptic feedback	‘… allowed me to get a feel for how to conduct these procedures, especially how I could position myself… how I may redo these next time.’ [MI 16]
		‘Hands‐on was essential to get an idea of the 3D positioning of hands and the amount of pressure needed to apply.’ [ME 54]
		‘It is helpful when you have a doctor watching and critiquing your technique.’ [ME 28]
		‘Able to ask questions to the educators whilst doing the hand on activity to ensure correct technique.’ [MI 22]
		‘Feedback on my technique allowed me to improve immediately.’ [ME 86]
		‘… gave me a good chance to use the equipment… also a good chance to see what should happen.’ [ME 49]
		‘Getting an idea of how the examination is performed and using equipment eg speculums feels like.’ [ME 52]
		‘… allowed me to practise my hand positioning and appreciate how the equipment works.’ [ME 59]
		‘I find women's health clinical skills in particular difficult to visualise and practice without hands‐on experience (for obvious reasons)… so the models really contextualised the previous learning.’ [ME 66]
	3.2 Observational learning	‘Seeing how senior doctors perform these procedures and the techniques they use is very useful. Their experience allows them to provide advice to make the procedure easier.’ [ME 43]
		‘I have been able to see which procedures are more uncomfortable for patients… how to have a good bedside manner… technique for intimate exams.’ [ME 32]
		‘I was able to see many of them… observe different people perform the same. This allowed me to explore and learn different techniques and taught me the most appropriate way I could perform the exam.’ [ME 37]
		‘Seeing how senior doctors perform these procedures and the techniques they use is very useful. Their experience allows them to provide advice to make the procedure easier.’
		‘Clinical observation… showed the full picture of what equipment I needed… how to position yourselves.’ [ME 49]
4. Scaffolded learning	4.1 Preparation	‘Prior to [the program] I had no observation in clinical practice. The video… was very useful in providing the basics… then actually getting to practise with this background made it easier.’ [ME 69]
		‘Enabled me to witness procedures I otherwise would not have at that point.’ [ME 46]
		‘… helped provide context as to why we do these procedures. It also taught me the equipment needed.’ [ME 61]
		‘… I wasn't blind coming into [the workshop]… the exams were familiar beforehand.’ [ME 62]
		‘… a lot of questions were answered… that clarified doubt before trying it all hands‐on in the practical session.’ [MI 20]
	4.2 Improved efficiency of in‐person workshop	‘It was useful to get an introduction before receiving more formal teaching, in order to make the most of the in‐person/hands‐on sessions.’ [ME 33]
		‘[It enabled] me to come to the workshops with questions. I could pay attention to the kinaesthetic aspect and not have to worry about the theoretical steps.’ [ME 64]
		‘Good to avoid wasting time [explaining knowledge during] practical sessions.’ [ME 68]
		‘… we already had the knowledge and thus had ample time to practise hands‐on.’ [ME 73]
		‘… we did not repeat the same information in the hands‐on session and focused primarily on practice.’ [ME 75]
5. Impact of online discourse	5.1 Indirect questioning	‘… the live Q&A allowed me to ask important questions.’ [ME 13]
		‘It was really easy to ask questions…’ [ME 12]
	5.2 Limitation of visual‐only learning	‘… learning the process step by step was very difficult without the active coordination and execution component, this was not retained.’ [ME 41]
		‘Cannot really have a feel of the force that the labour would impose and the tactile [nature] of the tools.’ [ME 4]
		‘… definitely not sufficient by itself as you need muscle memory.’ [ME 74]
	5.3 Limitation of filming	‘[Couldn't] really see what the demonstrator was doing.’ [ME 5]
		‘It wasn't very easy to see and follow…’ [ME 24]
	5.4 Difficult to engage focus	‘It was hard to concentrate… since the online workshop was held on a day of many other lectures… many of us would have zoomed out due to zoom fatigue.’ [ME 37]
		‘… online delivery made it difficult to engage with and retain.’ [ME 8]

### Low‐pressure simulation environments

A considerable theme to emerge from the data centred around low‐pressure simulation environments. Compared to a real‐life, real‐time clinical situation, removing patient considerations and time pressures allowed students to focus entirely on building their knowledge and skills [*1.1, 1.2*]. They favoured the opportunity to repeat the skill multiple times in succession with immediate expert feedback and troubleshooting [*1.3, 1.4*]. This aspect of the program greatly increased confidence for them to transition into clinical practice.

### Peer‐assisted learning

Students recognised the added benefits of learning by watching others through observation of different techniques and cumulative experience of mistakes and troubleshooting [*2.2*]. Medical and midwifery students engaged bi‐directionally to fill each other's gaps in pre‐existing knowledge and approach [*2.1*]. Questions directed toward each other were able to be shared with the broader group [*2.1*].

### Haptic learning

Hands‐on practice provided an opportunity to rehearse motor function, receive tactile feedback, familiarise themselves with equipment, and was an important aspect of learning, particularly for male students who felt a lack of natural access to female reproductive anatomy [*3.1*]. Furthermore, observation assisted students to put together all the steps required to perform a skill competently and compare different methods [*3.2*].

### Scaffolded learning

Students recognised the role of preparation to enhance learning and maximise opportunity during their in‐person workshop. Repeat exposure through various learning modalities provided foundational knowledge, context, and increased confidence prior to skills training [*4.1*]. Various responses detailed the importance of asking questions at multiple time points across a learning journey to clarify and solidify knowledge [*4.1*]. Consequently, the increased time devoted to hands‐on learning was greatly appreciated and students felt ready, asking higher order questions [*4.2*].

### Impact of online discourse

A significant volume of responses detailed the limitations of the live online demonstration, bringing to attention the single point focus of the filming [*5.3*], the notion of ‘Zoom^©^ fatigue’ [*5.4*], and the difficulty in translating visual knowledge to practical application over the medium [*5.2*]. Interestingly, the opportunity to ask experts questions indirectly, via the chat function, allowed students to be more boldly interactive and avoid feeling vulnerable [*5.1*].

## DISCUSSION

This study explores pedagogies that influence development of core obstetric and gynaecological clinical skills among the next generation of Australian doctors and midwives, and possibly future college trainees and Fellows. Low‐pressure simulation environments, peer‐assisted learning, haptic learning, scaffolded learning, and the impact of online discourse all play significant roles in how students learn and maximise their educational opportunities within a blended learning program (see Fig. 2).

Repetition is a key pedagogy, that Bruner describes as ‘…slow engagement with ideas… [that] builds into a critical mass when the student actually acquires the idea…’, and suggests that ‘…one should consciously design repetitive engagement into courses…’^12^ Within the blended program, there existed several opportunities for self‐paced discovery, contextualisation and clarification. While all sources led to a degree of learning, the theme of scaffolded learning promoted multiple sources to complement and enhance each other. This approach to learning creates building blocks toward achieving clinical proficiency.

Online resources provided students with learning materials that they could access at any point in their learning journey. The online demonstration and live Q&A along with the online resources, enhanced preparedness, and primed students to better absorb content when attending the in‐person workshop. This is consistent with positive outcomes using blended learning models incorporating video‐assisted learning materials across healthcare education (including specifically prior to performing pelvic examination), compared to standard lecture and simulation session alone.^13^, ^14^, ^15^ Synchronous interactive engagement, an effective pedagogical technique when teaching obstetrics and gynaecology content to medical students, was supplemented in the video component.^16^ Students were able to ask deidentified questions via the Zoom^©^ chat function immediately upon thought, and without interrupting the demonstrator. This process allowed students to avoid shame and fear associated with hierarchical barriers.^17^

However, teaching and learning via online discourse present several challenges. The phenomenon of ‘Zoom^©^ fatigue/burnout’ poignantly represents the unprecedented load of online learning students currently undertake. It has previously been described as ‘..the [constant, prolonged] need for attentiveness to nonverbal cues.’^18^ Suggested strategies to prevent this include limiting the use of videoconferencing technology or staggering breaks,^19^ although this is difficult for students in the setting of prescribed timetables, and instead the onus may fall on educators to make these considerations. Constructive criticism was received regarding the novice approach to filming the online demonstration and live Q&A. A smartphone camera was selected for its increased mobility, ease of use and accessibility. There may be a need for professionally trained technicians and multiple camera angles to further enhance the visuospatial representation of future demonstrations.

Among student cohorts, online‐facilitated education is broadly accepted and has high satisfaction rates, although participants recognise that it does not replace conventional in‐person teaching – it functions as an adjunct, not a substitute.^20^ While blended learning is not a new concept, increasing reliance on this approach places further scrutiny on its contribution to education.

Low‐pressure simulation environments develop confidence in students to perform clinical skills on real patients by first ‘practising without risk’.^21^ This serves as a bridge between online learning and clinical placement. Although patients generally have a favourable attitude toward students' involvement in their care,^22^ a significant barrier to learning is students not performing the skill effectively due to concern about patient discomfort or distress, and subsequently not achieving the desired outcome. Removing this risk and controlling for variables (acuity, patient, environment, equipment) allowed establishment of basic skills before moving onto secondary considerations. The ability to repeat examinations further consolidated skills, especially given the lack of opportunity to practise as a student such intimate procedures on real patients.

Peer‐assisted learning is a useful tool in clinical education.^23^ A variety of tutors taught in the workshops, ranging from final‐year medical students to consultants and senior midwifery academics. Not exclusively, the more junior a tutor, the more likely they are able to empathise with student difficulties, barriers to learning and assessment of pre‐existing knowledge.^24^ Furthermore, peer‐to‐peer discussion was profound during the in‐person workshop where midwifery and medical students worked together to share their different experiences and knowledge. Through observation and discussion, students practised bi‐directional critical enquiry, self and peer assessment and cooperated to troubleshoot tasks.^25^ Interestingly, this also took place during the online demonstration and live Q&A using the Zoom^©^ chat function, and probes further inquiry into the spontaneity and utility of online‐facilitated peer‐assisted learning.^26^

Motor learning is thought to primarily be driven by haptic components – both tactile (touch sensation) and proprioceptive (whole body coordination) – to continually refine movement control.^27^ This requires haptic feedback in the form of force feedback – ie experimental manipulation on models with a speculum and on digital vaginal examination was an important learning step to improve performance in the core clinical skills – but also augmented (externally provided) feedback.^28^ Tutors provided students with knowledge of their performance and were able to provide recommendations verbally or physically about body positioning, form, and technique.

However, motor learning also involves observational learning,^27^ which took place at multiple time points across the program: first at the online demonstration, followed by clinical‐based placement and finally at the workshop. Physical demonstration of the skill by more experienced clinicians provided a blueprint for movement strategies, spatial sequencing, timing, and dynamic movements for students to replicate and build into their own approach. Understandably, students were less exposed to a range of sensory outcomes during an online demonstration compared to the in‐person workshop.

### Limitations

The use of surveys may lack depth compared to conducting focus groups or interviews. However, surveys efficiently collect data from a larger number of participants. Sample size within the context of qualitative thematic analysis research is a divisive topic.^30^ We argue against using saturation as a proxy marker of significant sample size, especially where our author group has conducted deep, interpretive efforts and contextualisation of the dataset, rather than simple coding. Additionally, this study did not address long‐term evaluation of the program, or evaluation of an alternative sequencing of the in‐person clinical skills workshop (ie prior to, rather than during clinical‐based placement), of which both topics could be of interest to a future readership.

## CONCLUSION

In‐person teaching remains a cornerstone of obstetric and gynaecological clinical skills education, of which interprofessional simulation and clinical‐based placement are key components. Teaching via online discourse alone is not sufficient to completely replace and provide comparable learning outcomes but certainly plays an important role to prime students' learning and to maximise in‐person opportunities and resources. Our study reveals key pedagogies of a blended (online and in‐person) learning program, providing further evidence to support its ongoing utility as a feasible and warranted approach to learning.

## FUNDING

No funding was received for the publication of this study

## Supporting information


**Appendix 1.** Written survey (verbatim).
