# Supplementary material for: Blended (online and in‐person) Women’s Health Interprofessional Learning by Simulation (WHIPLS) for medical and midwifery students
Source: Aust N Z J Obstet Gynaecol. 2022 Apr 18;62(4):596–604. doi: 10.1111/ajo.13531 (PMC9544949; doi:10.1111/ajo.13531)
Supplement: Supplementary file 1 — Appendix 1. Written survey (verbatim). [file AJO-62-596-s001.docx]

**Appendix 1:** Written survey (verbatim)

| **WHIPLS (online and in-person) evaluation form**  I am a: Medical Student ☐ Midwifery Student ☐   1. Please allocate what percentage of your knowledge related to the core clinical skills (to a total of 100%) you feel you have gained from the following delivery methods:  - Pre-reading and video lectures - Online demonstration with live Q&A - Clinical observation and assisting - Hands-on practice with training models  1. In a short paragraph, please explain how the online demonstration with live Q&A contributed (or not) to your core clinical skills. 2. In a short paragraph, please explain how the clinical observing and assisting (in birth units, clinics etc.) contributed (or not) to your core clinical skills. 3. In a short paragraph, please explain how the hands-on practice with training models in the workshop contributed (or not) to your core clinical skills. 4. In a short paragraph, how did prior observation of practical skills (videos, live zoom demonstration, observing clinical practice) enhance or diminish your hands-on learning experience with the training models? |
| --- |
